# Fibre Optic Method for Detecting Oil Fluorescence in Marine Sediments

**DOI:** 10.3390/s25010173

**Published:** 2024-12-31

**Authors:** Emilia Baszanowska, Zbigniew Otremba, Maria Kubacka

**Affiliations:** 1 Department of Physics, Gdynia Maritime University, Morska 81-87, 81-225 Gdynia, Poland; z.otremba@wm.umg.edu.pl; 2 Department of Operational Oceanography, Maritime Institute, Gdynia Maritime University, ul. Roberta de Plelo 20, 80-848 Gdańsk, Poland; mkubacka@im.umg.edu.pl

**Keywords:** sediments, seawater, oil in sediments, oil fluorescence, oil detection, fibre optic, excitation–emission spectra

## Abstract

The aim of this study is to verify the possibility of detecting oil in the bottom sediment using a fibre optic system. The presence of oil is assessed on excitation–emission spectra obtained from spectral fluorescence signals of the sediment sample. A factory spectrofluorometer coupled with an experimental fibre optic measurement system was used. During the determination of spectra, the fibre optic system is set at a 45° angle to the sediment surface and placed above its surface. The light exciting the fluorescence and the light emitted by the sediment are transmitted in a combined bundle of fibre optic threads. The analysis of excitation–emission spectra of sediments contaminated with oil shows variability of the shapes of fluorescence spectra depending on the type and degree of oil contamination, which indicates the feasibility of the sensor design for detecting oil in the sediment in situ.

## 1. Introduction

Sediments play a key role in aquatic ecosystems by providing nutrients and serving as habitats for aquatic organisms [1,2,3,4]. However, human activities lead to the accumulation of toxic substances, such as oil and heavy metals, which are persistent and toxic environmental pollutants. Petroleum hydrocarbons found in aquatic environments include primarily alkanes, olefins, and aromatic compounds [5]. Due to their low solubility in water, oils attach to suspended particulate matter, persisting for long periods in seabed sediments and adversely affecting benthic organisms [6,7,8]. Petroleum hydrocarbons and heavy metals accumulate in sediments, impacting ecosystems through bioaccumulation and incorporation into the food chain, posing a threat to organisms and causing habitat changes [9,10].

Studies on sediment contamination are crucial, as marine sediments record long-term anthropogenic impacts, enabling the assessment of water pollution levels. Sediments also play a role in the biogeochemical cycle of heavy metals and can act as a source of secondary pollution. The contamination of sediments with petroleum hydrocarbons poses a significant risk to coastal marine ecosystems, resulting in plant and animal diseases, species extinction, and changes in ecosystem structures.

Substances from various oil discharges can enter seawater and bottom sediments as a result of physical, chemical, and biological processes [11,12,13,14]. The most important mechanisms enhancing oil adsorption to sediments include leaks from shipwrecks [15,16]. Over the years, metal parts of vessels corrode, which leads to the release of residual fuels, lubricating oils, and other chemical substances from cargo compartments, tanks, or mechanical systems. A wreck on the seabed may become disintegrated by currents, storm surges, or human activity (e.g., trawling or even blowing up as an underwater obstacle), which may release harmful substances into the surroundings. The selection of the appropriate methodology for detecting the presence or determining the concentration of hydrocarbons included both the method and frequency of sampling, as well as laboratory analysis techniques. Typical analytical methods used to assess hydrocarbon content are gravimetry (determining non-soluble substances), UV and IR spectroscopy, gas chromatography, and gas chromatography coupled with mass spectrometry [17]. All these methods require complex laboratory procedures and are time-consuming. Additionally, the process of extracting petroleum substances from water requires a large volume of samples, which adds further difficulty to the analysis.

To assess the release of oil substances into seawater from sediments, studies were conducted on the impact of sediments near the wreck s/s *Stuttgart* on near-seabed water [18]. The presence of oil contamination in seawater can be detected by analysing the peak distribution in the total fluorometric spectrum (Ex–Em) in the ultraviolet range [19,20]. Laboratory tests including fluorometric analyses are carried out in three stages, during which the excitation–emission spectra (EEMs), fluorescence intensity, and fluorometric index (FI) are determined. They aim to determine whether hydrocarbons originating from the wreck lying in the seabed sediments affect the state of seawater in its immediate vicinity. The results revealed the presence of oil substances both in the sediments and in the near-seabed water at three out of six analysed sampling points, which confirms the spatial heterogeneous distribution of these compounds. The analyses of sediments based on laboratory tests require the collection of sediments and seawater from the seabed, their transportation to the laboratory, and finally, the analyses. However, it would be highly advantageous to indicate the presence of oil substances in sediments directly in the marine environment.

Therefore, the goal of the study is to detect oils in seabed sediments using an experimental method based on a system of optical fibres connected to a spectrofluorometer. The study involved the determination of excitation–emission matrices (EEM) for sediment (free of oil) from the seabed, as well as sediments artificially contaminated with oils. The resulting EEM spectra and the wavelength-independent fluorescence maxima (λ_Ex_/λ_Em_) allowed the distinction of differences in the spectra of seabed sediments and those artificially contaminated with oils. The research conducted indicates that the proposed method for the detection of oil in sediments is promising for the use of an optical fibre system in relation to in situ measurements.

## 2. Materials and Methods

Samples for the measurements were taken from the point located in a region of the Baltic Sea known as the Gulf of Gdańsk at location 18°36′35″ E 54°33′22″ N.

### 2.1. Sediment Sample

Surface sediments were sampled using a van Veen grab sampler (0.1 m^2^, 6 kg), lowered on a steel cable from a hydraulic winch. The sampler collected sediments from the seafloor to a depth of 18 m, penetrating the sediment to a depth of 10–20 cm (depending on soil conditions). After retrieval, the samples were placed in plastic zip-lock bags on the research vessel’s deck.

To assess the physicochemical properties of sediments, the sediment samples were dried using the natural air grain drying method. Next, the characteristics of the value distribution parameters were determined using an automated particle analyser (Morphologi G3). The values of the distribution parameters are presented in Table 1. The interstitial water from the sediment was analysed for the presence of oil substances using the EEM spectra and fluorometric index FI [18]. The sediments sampled were qualified as free of oil.

### 2.2. Near-Seabed Seawater Samples

Near-seabed water was collected at the sediment sampling location from a depth of 18 m. To assess the hydro-physical conditions indicating the presence of petroleum substances in sediments and near-seabed seawater, water parameters were measured using a CTD 115 M probe from aboard the motorboat *IMOROS 2* (Maritime Institute of the Gdynia Maritime University) on 23–24 June 2023. The gathered data on the physicochemical properties recorded (salinity, temperature, dissolved oxygen (DO) and PH) are presented in Table 2 with the minimum to maximum and the average values and the standard deviation (SD) included. At the selected seawater sampling point, the mean values of the parameters measured were salinity 7.64 PSU, temperature 4.68 °C, dissolved oxygen DO 10.32 mg/L, and PH 7.63.

### 2.3. Oil Samples

- Heavy fuel oil—HFO—density at 15 °C: 981 kg/m^3^, viscosity at 50 °C: 2800 mPa·s,
- Crude oil—*Petrobaltic*—extracted from the Baltic Sea shelf, light crude, with the American Petroleum Institute (API); gravity: 43–44°; sulphur content: 0.12%.

### 2.4. Measurement and Apparatus

**Figure 1:**
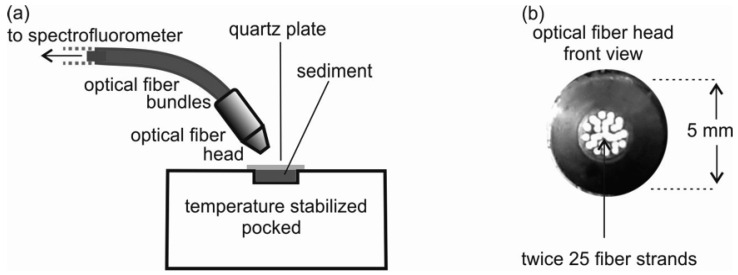
An optical fibre used to detect oil in sediments: the outside connection with the spectrofluorometer (**a**); the front view of the optical fibre head (**b**).

The optical fibre produced by Lumex Ltd., St. Petersburg, Russia employing a spectrofluorometer (Hitachi F-7000 FL, Hitachi, Ltd.; Tokyo, Japan), was used for oil detection in sediments. The optical fibre system was conducted outside the spectrofluorometer. Using optical fibre (Figure 1a) for UV light excitation, as well as in the measurements of fluorescence intensity, the excitation–emission spectra (EEMs) of oil-free sediment and sediment samples artificially polluted with oil were determined.

#### 2.4.1. Optical Fibre System

The optical fibre consists of 2 × 25 fibre strands of 0.1 mm in diameter each (25 fibre glasses transmit light that excites fluorescence, and the other 25 transmit light emitted by oil), as presented in Figure 1b. The fluorescence of the sediment was excited by light from a fibre optic head located above the surface of the oil-contaminated sediments at a distance of 3 mm (Figure 1). The measurements were performed at an angle of 45° to the sediment surface (Figure 1a).

#### 2.4.2. Spectrofluorometer

A Hitachi F-7000 FL spectrofluorometer (Hitachi, Ltd; Tokyo, Japan) was used to determine the EEMs. The excitation wavelength was changed from 240 to 420 nm with an excitation wavelength interval of 5 nm. The emission wavelength was changed from 260 to 540 nm with a 5 nm emission interval, a 20 nm excitation slit, and a 20 nm emission slit. The integration time was 0.5 s, and the photomultiplier tube voltage was 400 V. During the measurements, the temperature of the fluorometer was stabilised at about 20 °C.

## 3. Results

**Figure 2:**
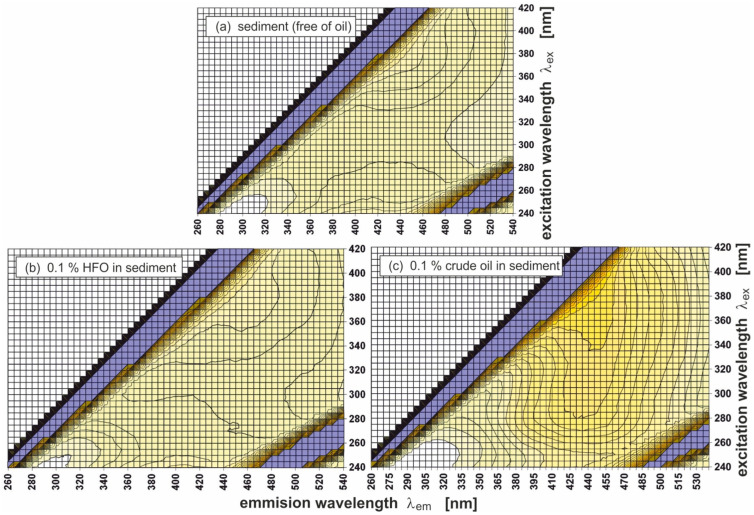
Excitation–emission fluorescence spectra of oil-free sediment (**a**) and the same sediment artificially polluted with two types of oil (**b**,**c**).

Figure 2 presents 2D EEMs for an oil-free sediment sample (a) and sediment samples polluted with two kinds of oil—heavy fuel oil and crude oil, respectively: 0.1% heavy fuel oil (HFO) in the mass of sediment (b) and 0.1% of crude oil (c). The EEMs of the sediments were determined using an optical fibre connected to a spectrophotometer, utilising a set of excitation and corresponding emission wavelengths. The EEM method allows for identifying characteristic fluorescence maxima of fluorescing components in sediment samples. These maxima were determined using the wavelength-independent fluorescence parameter (λ_Ex_/λ_Em_), described by the maximum fluorescence for the excitation wavelength corresponding to the emission wavelength. Sediments free of oil and sediments polluted with oil are indicated by the presence of specific peaks (λ_Ex_/λ_Em_):

(a) 250/414 and 370/450 for sediment free of oil,
(b) 370/460 and 270/400 for polluted sediments with HFO (0.1%),
(c) 300/435 and 355/445 for polluted sediments with crude oil (0.1%).

The data for parameter (λ_Ex_/λ_Em_) are presented in Table 3.

The analysis of determined (λ_Ex_/λ_Em_) for oil-polluted sediments polluted by oil in relation to sediments free of oil indicate the presence of (λ_Ex_/λ_Em_) = 270/400, 300/435, and 355/445, which is responsible for oil fluorescence.

Figure 2 indicates the changes in the EEMs shape of sediment in the case of even very low amounts of oil in sediment. The EEMs in Figure 2 and some of the following figures show fragments responsible for light scattering (when λ_em_ = λ_ex_ and λ_em_ = 2 × λ_ex_).

**Figure 3:**
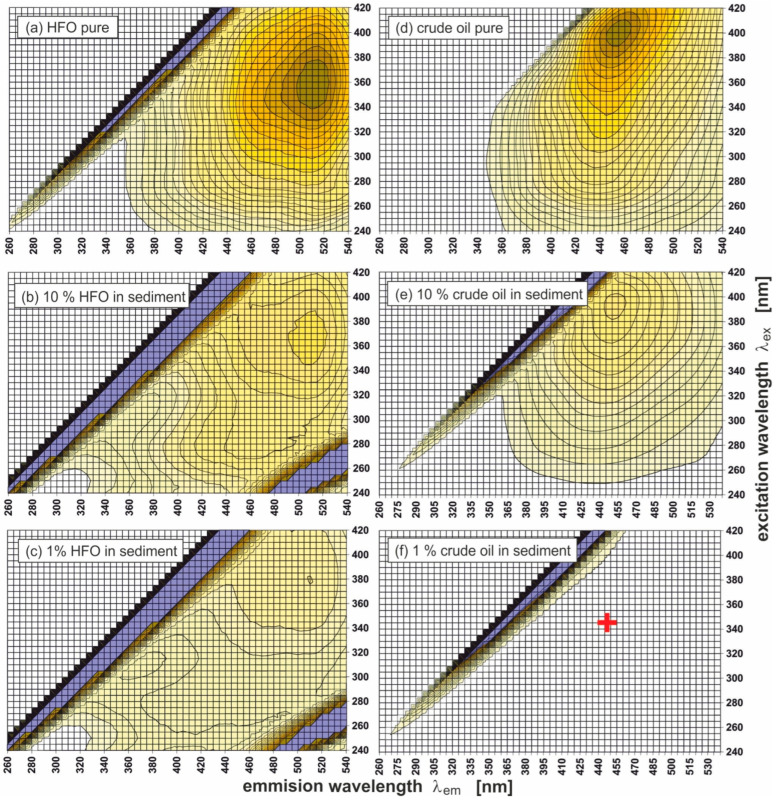
Excitation-emission spectra (EEMs) of two types of oil (upper graphs) and spectra of oil-polluted sediment (the same as in Figure 2 but more polluted). The graphs on the left have the same scale relative to the HFO graph, those on the right to the crude oil graph. The cross in the graph (**f**) indicates the peak location.

Figure 3 presents 2D EEMs for two types of oil: heavy fuel oil (HFO) (Figure 3a) and crude oil (Figure 3d) and sediments polluted with two oils with various oil contents, respectively: HFO 10% (b), HFO 1% (c), crude oil 10% (e), and crude oil 1% (f). The EEMs presented on the left (Figure 3a–c) have the same scale relative to the HFO graph, while those on the right to the crude oil graph (Figure 3d,e). The data for (λ_Ex_/λ_Em_) determined for those kinds of oil and different oil amounts (1% and 10%) adsorbed by sediments are presented in Table 4.

Pure oils and sediments polluted with these oils are characterised by the presence of specific peaks (λ_Ex_/λ_Em_):

(a) 360/510 for pure HFO,
(b) 290/465 and 370/510 for sediments polluted with HFO (10%),
(c) 310/370 and 380/510 for sediments polluted with HFO (1%),
(d) 400/455 pure crude oil,
(e) 390/448 for sediments polluted with crude oil (10%),
(f) 380/430 (marked by a cross in Figure 3f) for sediments polluted with crude oil (1%).

**Figure 4:**
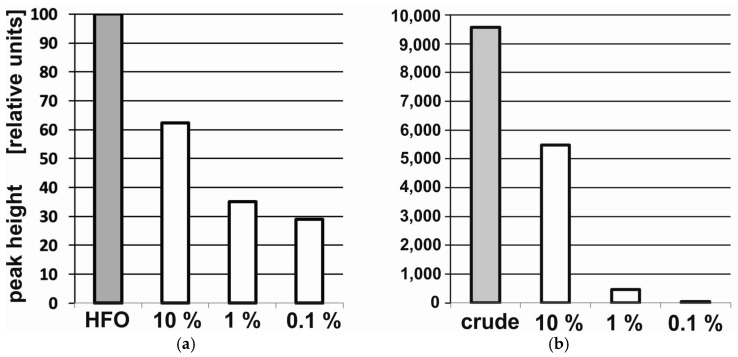
The height of the main fluorescence peaks for sediment artificially polluted with an oil of the chosen types: heavy fuel oil (HFO) (**a**), crude oil (**b**).

The maximum peak in Figure 3f is invisible, as the intensity of fluorescence for a 1% amount of oil added to sediment is too low in relation to the intensity of fluorescence for pure crude oil presented in Figure 3d (Figure 3d–f are plotted on the same scale). It should be mentioned that the peak in Figure 3f is one order higher than the peak in Figure 3c (as shown in Figure 4). In Figure 3d (pure crude), the scattering effect is not visible, because in this spectrum, the fluorescent light dominates the scattered light.

The difference in fluorescence intensity for the two types of oil to the amount of oil in the sediment is presented in Figure 4. Analysis of the relationship between fluorescence intensity and the type of oil indicates that the intensity of the HFO fluorescence values in the sediment are much lower than in the case of crude oil. On the other hand, even only a 0.1% share of crude oil in the sediment can be detected.

## 4. Discussion

**Figure 5:**
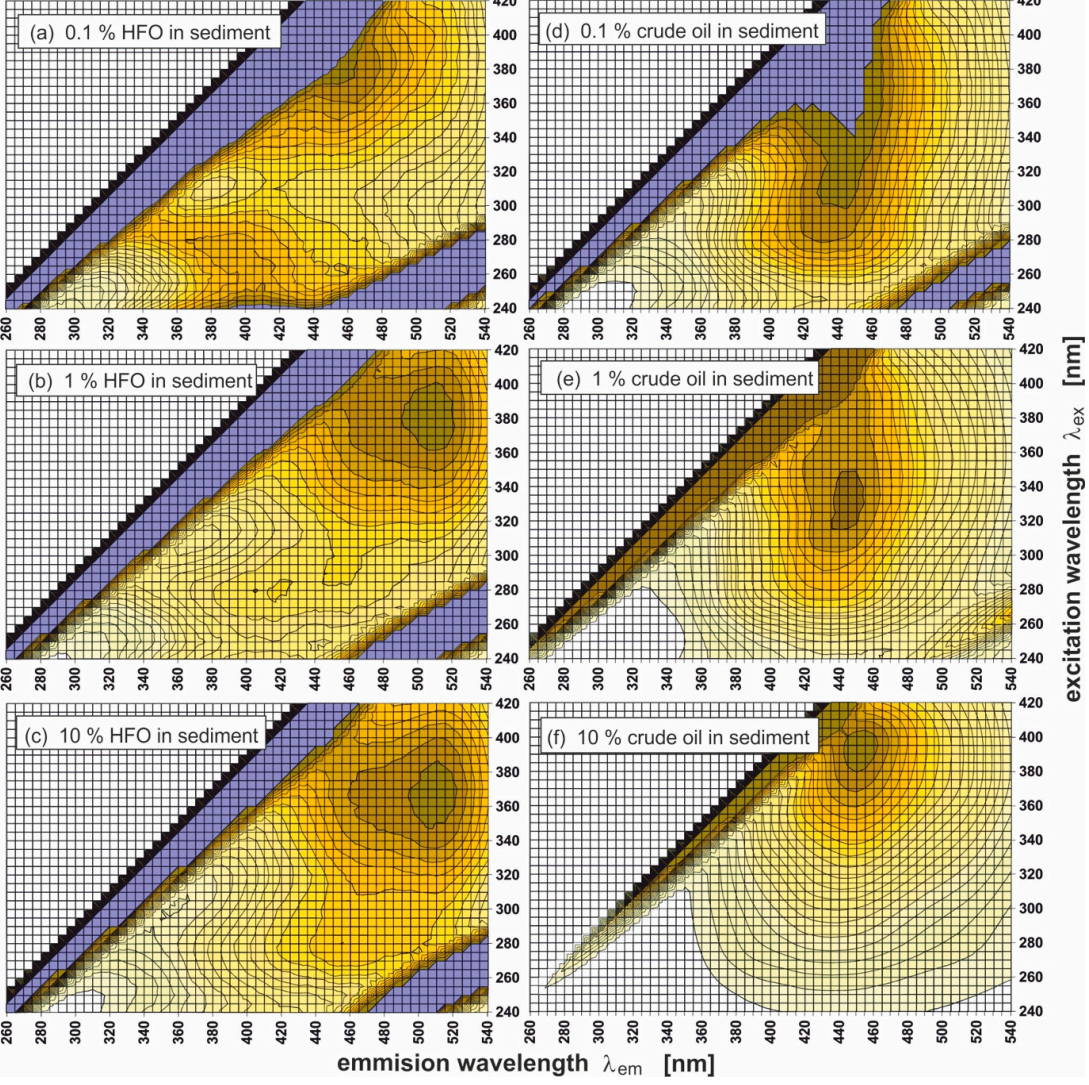
Normalised EEM spectra for sediments polluted with two different oils at various oil concentrations, respectively: HFO 0.1% (**a**), HFO 1% (**b**), HFO 10% (**c**), crude oil 0.1% (**d**), crude oil 1% (**e**), and crude oil 10% (**f**). Normalisation was performed for both types of oil and each predetermined amount.

To analyse the impact of oil present in the sediment in different amounts on the changes of the EEMs, normalisation of the EEM spectra was performed for the oils of both kinds and in each pre-determined amount (Figure 5). The data for the parameter (λ_Ex_/λ_Em_) determined for the normalised EEMs, for different kinds of oil, and different amounts of oil added (1% and 10%) to the sediments are presented in Table 5. For a particular kind of oil and amount of oil added to the sediment, specific peaks were determined (λ_Ex_/λ_Em_):

(a) 270/390 and 380/450 for sediment polluted with HFO (0.1%),
(b) 280/420, 290/450, and 380/510 for sediment polluted with HFO (1%),
(c) 285/460 and 370/510 for sediment polluted with HFO (10%),
(d) 310/440 for sediment polluted with crude oil (0.1%),
(e) 330/450 for sediment polluted with crude oil (1%),
(f) 390/450 for sediment polluted with crude oil (10%).

The position of the peaks (λ_Ex_/λ_Em_) depends on the degree of sediment contamination. The reason for this dependence lies in the interaction of sediment particles with the oil. The nature of this phenomenon has not been fully explained yet. This effect makes it difficult to decide on the excitation wavelength and emission wavelength used in the probable oil-in-sediment sensor. In the case of the sensor detecting HFO in sediment, the excitation wavelength varies between 370 and 380 nm (an increase in oil concentration causes a shift of the excitation wavelength towards shorter wavelengths). The emission wavelength, on the other hand, shifts towards longer wavelengths with increasing concentration in the range from 450 nm to 510 nm. However, in the case of Baltic crude oil, it would be necessary to assume a range of 330–380 nm for excitation and a level of 405 nm for emission. Therefore, it can be concluded that a given sensor should be dedicated to detecting a specific type of oil in the sediment (future studies using different sediments and different types of oil would be needed to solidify the above conclusions).

**Figure 6:**
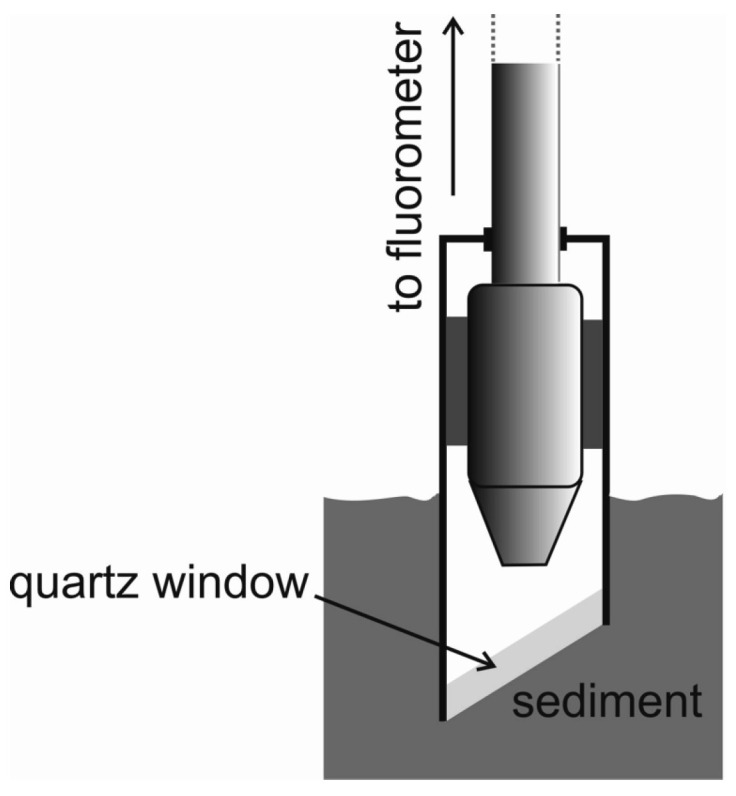
A general scheme of the operation of an oil detector in seabed sediments.

Figure 6 shows that the quartz sensor window must be tilted relative to the light coming from the fibre optic head, which eliminates light reflection. If the window was perpendicular to the fluorescence–excitation light, the fluorescence light detector would be dazzled. It should be highlighted that the knowledge about the optimal positioning of the fibre optic head was obtained through experimental analysis. The method, compared to other fluorometric methods, has a unique value. It is dedicated to detecting oily substances in sediments through contact using a measuring head. Other methods require preliminary laboratory processing.

In summary, our approach introduces a significant innovation by integrating optical fibre excitation and emission paths. This advancement enables direct, in situ measurements of oily substances in sediments, eliminating the need for labour-intensive sample preparation typically required in traditional methods. Additionally, the application to new types of samples further demonstrates the versatility and practical applicability of this enhanced technique. These modifications mark a notable progression in the method’s development.

## 5. Conclusions

The objective of this study was to verify a potential method for detecting oil in seabed sediments using a system of optical fibres integrated with a spectrofluorometer. Tests for the excitation of samples outside the spectrofluorometer using this optical fibre system and registration of excitation–emission spectra (EEMs) using a fluorometer were performed for oil-free sediment from the seabed and on samples of sediment artificially contaminated with oils. Analysis of the EEM spectra and the fluorescence maximum parameter allowed the identification of differences between the spectra of natural sediments and those contaminated with oils. Moreover, differences in EEMs were detected in relation to oils of different types. The results suggest that the proposed method is a step forward in research towards the practical application of the optical fibre system in in situ detection of oil in sediment.

## Figures and Tables

**Table 1:** Characteristics of the value distribution parameters in a sediments sample. D[n, 0.1]—10% of the particles are smaller than this diameter. D[n, 0.5]—half of the particles are smaller than this diameter and half are longer. D[n, 0.9]—90% of the particles are smaller than this diameter.

Characteristics of the Value Distribution Parameter	Min.	Max.	Mean ± SD	D[n, 0.1]	D[n, 0.5]	D[n, 0.9]
Diameter, µm	0.54	452.24	3.34 ± 6.65	0.68	1.56	7.34
Circularity	0.022	1.000	0.809 ± 0.171	0.547	0.860	0.958
Convexity	0.293	1.000	0.983 ± 0.043	0.893	0.985	0.996
Shape coefficient	0.024	1.000	0.719 ± 0.157	0.499	0.730	0.894
Solidity	0.107	1.000	0.969 ± 0.069	0.801	0.961	0.993

**Table 2:** Biogeochemical parameters of the near-seabed water at the sediment sampling location in the Gulf of Gdańsk.

Near-Seabed Water Level [m]	Salinity [PSU]		Temperature [°C]		DO [mg/L]		PH	
Min–Max	Min–Max	Mean ± SD	Min–Max	Mean ± SD	Min–Max	Mean ± SD	Min–Max	Mean ± SD
18–18.5	7.61–7.65	7.64 ± 0.007	4.69–4.97	4.68 ± 0.070	10.24–10.40	10.32 ± 0.03	7.61–7.65	7.63 ± 0.01

**Table 3:** Major fluorescent peaks with their wavelength-independent fluorescence maxima (λ_Ex_/λ_Em_) for an oil-free sediment sample and sediment samples polluted with HFO and crude oil in the amount of 0.1%.

Ex_max_ [nm] ± 5 [nm]/Em_max_ [nm] ± 5 [nm]					
Sample	Peak 1	Peak 2	Oil Peak	Oil Peak	Oil Peak
sediment (free from oil)	250/414	370/450			
0.1% of HFO in sediment		370/460	270/400		
0.1% of crude oil in sediment				300/435	355/445

**Table 4:** Major fluorescent peaks with their wavelength-independent fluorescence maxima (λ_Ex_/λ_Em_) for pure oils: HFO and crude oil, respectively, and for sediment samples polluted with HFO and crude oil with the oil contents of 10% and 1%.

Ex_max_ [nm] ± 5 [nm]/Em_max_ [nm] ± 5 [nm]					
Sample	Sediments Peak 1	Sediments Peak 2	Oil Peak	Oil Peak	Oil Peak
HFO (pure)	-	-	-	360/510	-
crude oil (pure)	-	-	-	-	400/455
10% of HFO in sediment	-	-	290/465	370/510	-
10% of crude oil in sediment	-	-	-	-	390/448
1% of HFO in Sediment	-	-	310/370	380/510	-
1% of crude oil in sediment	-	-	-	-	380/430

**Table 5:** Major fluorescent peaks with their wavelength-independent fluorescence maxima (λ_Ex_/λ_Em_) for normalised EEMs for pure oils: HFO and crude oil, respectively, and sediment samples polluted with HFO and crude oil constituting 10% and 1% of the samples.

Sediments Polluted with Oil	Oil Peak	Oil Peak
0.1% of HFO	270/390	380/450
0.1% of crude oil	310/440	
1% of HFO	280/420 290/450	380/510
1% of crude oil	330/450	
10% of HFO	285/460	370/510
10% of crude oil	390/450	

## Data Availability

The datasets used and analysed during the current study are available from the corresponding author on reasonable request.
